# Methods of defining the non-inferiority margin in randomized, double-blind controlled trials: a systematic review

**DOI:** 10.1186/s13063-017-1859-x

**Published:** 2017-03-07

**Authors:** Turki A. Althunian, Anthonius de Boer, Olaf H. Klungel, Widya N. Insani, Rolf H. H. Groenwold

**Affiliations:** 1 Division of Pharmacoepidemiology and Clinical Pharmacology, Utrecht Institute of Pharmaceutical Sciences, Utrecht University, PO Box 80082, 3408 TB Utrecht, The Netherlands; 2 Julius Center for Health Sciences and Primary Care, University Medical Center Utrecht, Huispost Str. 6.131, 3508 GA Utrecht, The Netherlands

**Keywords:** Non-inferiority trials, Non-inferiority margin, Methodology, Drug regulation

## Abstract

**Background:**

There is no consensus on the preferred method for defining the non-inferiority margin in non-inferiority trials, and previous studies showed that the rationale for its choice is often not reported. This study investigated how the non-inferiority margin is defined in the published literature, and whether its reporting has changed over time.

**Methods:**

A systematic PubMed search was conducted for all published randomized, double-blind, non-inferiority trials from January 1, 1966, to February 6, 2015. The primary outcome was the number of margins that were defined by methods other than the historical evidence of the active comparator. This was evaluated for a time trend. We also assessed the under-reporting of the methods of defining the margin as a secondary outcome, and whether this changed over time. Both outcomes were analyzed using a Poisson log-linear model. Predictors for better reporting of the methods, and the use of the fixed-margin method (one of the historical evidence methods) were also analyzed using logistic regression.

**Results:**

Two hundred seventy-three articles were included, which account for 273 non-inferiority margins. There was no statistically significant difference in the number of margins that were defined by other methods compared to those defined based on the historical evidence (ratio 2.17, 95% CI 0.86 to 5.82, *p* = 0.11), and this did not change over time. The number of margins for which methods were unreported was similar to those with reported methods (ratio 1.35, 95% CI 0.76 to 2.43, *p* = 0.31), with no change over time. The method of defining the margin was less often reported in journals with low-impact factors compared to journals with high-impact factors (OR 0.20; 95% CI 0.10 to 0.37, *p* < 0.0001). The publication of the FDA draft guidance in 2010 was associated with increased reporting of the fixed-margin method (after versus before 2010) (OR 3.54; 95% CI 1.12 to 13.35, *p* = 0.04).

**Conclusions:**

Non-inferiority margins are not commonly defined based on the historical evidence of the active comparator, and they are poorly reported. Authors, reviewers, and editors need to take notice of reporting this critical information to allow for better judgment of non-inferiority trials.

**Electronic supplementary material:**

The online version of this article (doi:10.1186/s13063-017-1859-x) contains supplementary material, which is available to authorized users.

## Background

Non-inferiority trials are a relatively new design of randomized clinical trials, which are increasingly being published [1]. Non-inferiority trials are conducted to investigate whether the efficacy of a novel (test) drug is not worse than that of an active comparator according to a predefined non-inferiority margin [2–4].

There is no consensus on the preferred method for defining the non-inferiority margin. Regulatory guidelines recommend defining the margin based on a comprehensive review of the historical evidence of the efficacy of the active comparator (mainly against placebo) [2, 5–7]. Only the US Food and Drug Administration (FDA) draft guideline recommends explicit methods on how to define the margin based on clinical and statistical considerations [2]. Other regulatory guidelines provide more general recommendations [5–7]. The impact of these guidelines in general, and the FDA draft guideline in particular, on the choice of non-inferiority margin is unknown. Most of the previous reviews on the methodological quality of published non-inferiority trials did not provide data on whether, and how, the historical evidence of the active comparator was used to define the margin [8–12].

The extension of the Consolidated Standards of Reporting Trials (CONSORT) Statement for reporting non-inferiority and equivalence randomized trials was published in 2006 to improve the quality of reporting non-inferiority and equivalence trials [13, 14]. This extension recommends that the margin and how it relates to the effect of the active comparator in any placebo-controlled trial should be noted. Three systematic reviews on non-inferiority trials found no significant impact of this statement on reporting the rationale for the choice of the margin in a before-after comparison [9, 10, 12]. None of these reviews provide details about the endorsement of the CONSORT Statement, and whether the reporting of the rationale for the choice of the margin was different in CONSORT-endorsing journals versus non-CONSORT-endorsing journals.

The aim of this systematic review was to identify the methods that are being used to define the non-inferiority margin in the published non-inferiority trials, and whether the margin is defined based on methods other than the historical evidence of the active comparator.

## Methods

### Study selection and search strategy

A systematic PubMed search was conducted for all published randomized, double-blind, non-inferiority trials. The search included the period January 1, 1966, to February 6, 2015, using (Noninferiority [Title/Abstract] AND double-blind study [MeSH Terms]) as search items, and it was restricted to randomized controlled trial, humans, and English-language publications. We targeted non-inferiority trials that could potentially have had an impact on drug regulatory decisions. Therefore, we excluded the following studies: phase I studies, pharmacodynamic studies, pharmacokinetic studies, bioequivalence studies, open-label and single-blind studies, studies with a non-pharmacological intervention as the experimental treatment, subgroup analysis, post hoc analysis, secondary analysis, extension studies of previously published studies, and published protocols.

### Methods of defining the non-inferiority margin

The non-inferiority margin is used to assess whether the test drug will preserve what is considered a clinically significant fraction of the effect of the active comparator. To that aim, historical evidence on the active comparator from (a meta-analysis of) placebo-controlled (and/or active controlled trials) is used [2, 15–17]. The point-estimate and the fixed-margin methods are methods of analyzing non-inferiority where the margin is defined based either on the effect estimate from the historical evidence or the limit of the confidence interval of the effect estimate that is the closest to the null effect. In the point-estimate method, the fraction of the effect estimate that is considered clinically significant is determined based on clinical judgement [15]. This fraction is then called the preserved fraction. The margin represents the remaining fraction of the point estimate of the active comparator that stakeholders are willing to lose at a cost of gaining other advantages that are claimed to be offered by the test drug (such as advantages in terms of safety or administration) [2, 15–18].

In the point-estimate method, the margin is defined based on the effect estimate, which does not capture the variance of the effect estimates of the active comparator from the past trials, and may not reflect the placebo-controlled effect of the active comparator that is expected to be present in the non-inferiority trial if a placebo arm were included [2, 15, 16]. This may lead to an estimation of a margin that is either too lenient or too strict. On the other hand, the fixed-margin method, the method that is recommended by the FDA, takes this into account by defining the margin based on the smallest effect size of the active comparator from the past trials (as expressed by the confidence limit that is closest to the null effect) [2, 15, 16].

The margin that was defined based on the pooled effect estimate in the point-estimate method can be used in another method of analysis of non-inferiority trials which is called the synthesis method. The difference between the point-estimate method and the synthesis method is that in the latter the variance of the effect estimates of the active comparator is incorporated in the analysis of non-inferiority (not in setting the margin) [2, 15, 16].

For this review, if the margin was not defined according to one of the aforementioned methods, the method that was used to define the margin was classified as “other methods”. A further subclassification of other methods was provided based on the type of general justification that was stated in each article for defining the margin. The method was considered “not reported” when the method of defining the margin was not stated in the article.

### Data extraction and assessment

The search in PubMed and the screening of each title and abstract was conducted by TA to identify eligible studies. Based on the full texts of eligible studies, the following were extracted from each article: (1) report characteristics: authors, the published journal, the date of publication, the number of trials in each article, the test and the active comparator, the indication, and the chosen outcome for analyzing non-inferiority; (2) information about the margin: the predefined non-inferiority margin(s), the choice and motivation of the margin, and the preserved fraction. A random sample of 10% of the total number of the included articles was also reviewed by AdB, OK, and RG to assess interobserver reliability. Disagreement occurred in one article with regard to the type of the method that was used to define the margin (kappa = 0.95). When available, the online supplementary materials of each article were checked. Additionally, the cited references were checked when the description of the choice of the margin was not clear. Discussions to reach consensus were held among the research team members to give a final classification of the method when the description of the choice of the margin was not clear in the original full text.

The endorsement of the CONSORT Statement was checked in the “Instructions for Authors” section in each journal. When reporting non-inferiority trials, the original CONSORT Statement recommends reading the extension of the CONSORT Statement for non-inferiority and equivalence trials [13, 14, 19]. A journal was classified as a CONSORT-endorsing journal if it endorses either the CONSORT Statement or the International Committee of Medical Journal Editors’ (ICMJE) uniform requirements for manuscripts submitted to biomedical journals [20].

We classified the journals into high- or low-impact journals based on the impact factor of each journal in the year of publishing the non-inferiority trial. The impact factor of each journal was collected from InCites™ Journal Citation Reports® (JCR) [21]. Journals with an impact factor of ten or more were classified as high-impact journals. For journals that do not have impact factors in the year of publication of the non-inferiority trial, the classification was based on the impact factor of the preceding or the succeeding year depending on the availability of the impact factor. The impact factors of two journals, *SKINmed Dermatology for the Clinician* and *Infectious Diseases in Obstetrics and Gynecology*, were not provided neither by JCR nor by their original websites. Thus they were considered as low-impact journals.

The impact of the FDA draft guidance on the use of the fixed-margin method, the recommended method by the FDA, was checked before and after the publication of this guidance in 2010 (articles that were published in and before 2010 compared to those published in 2011 and thereafter). The FDA draft guideline was chosen because it is the last published regulatory guideline, and it is the only one that provides explicit methods to define the margin. The adherence with the CONSORT Statement in reporting the rationale for the choice of the margin was analyzed in articles that were published in and before 2006 compared to those published in 2007 and thereafter. The reporting of the rationale was also analyzed in CONSORT-endorsing versus non-CONSORT-endorsing journals, and in high- versus low-impact journals.

### Study outcomes

The primary outcome of the study was the number of margins that were defined by “other methods”. This number was compared to the number of margins that were defined by the historical evidence of the active comparator (ratio), and was assessed for a time trend. The secondary outcome was the number of margins with unreported methods, which was compared to the number of margins with reported methods (regardless of the method type), and evaluated for a time trend. The odds of not reporting the method of defining the margin (margins with unreported methods/margins with reported method) were assessed for three predictors: the publication of the extension of the CONSORT Statement in 2006 (before versus after), the endorsement of the CONSORT Statement by the journals (endorsing versus non-endorsing), and the journals’ impact factor (high versus low). Finally, the odds of using the fixed-margin method to define the margin were assessed before versus after the publication of the 2010 FDA draft guidance.

The analyses were conducted based on the margin that was used to analyze the primary outcome in each trial (i.e., one margin per trial). If more than one margin was used in a trial, and it was not clear which margin was for the primary analysis, the one that was used for the sample size calculation was used. If both margins were used in the sample size calculation, the margin that was stated first was used. Even if the analysis of non-inferiority was a secondary outcome in a trial (i.e., superiority was the primary outcome), it was considered in this review. A sensitivity analysis was performed for all outcomes using all margins that were used in the included trials.

### Statistical analysis

A descriptive statistical summary was provided for the methods used to define the margin and for the preserved fractions. Poisson regression was used to test whether the number of margins that were defined based on other methods was different compared to the number of those defined based on the historical evidence of the active comparator, and whether the ratio of these numbers has changed over the years (the margins with unreported methods were excluded from this model). The change was determined by assessing the interaction between method and the year of publication. A separate Poisson model was also used to estimate the ratio of number of margins with unreported methods to the number of margins with reported methods (margins defined based on the historical evidence and other methods). The year of publication was added as an independent variable and the interaction with the method variable was assessed to determine whether the ratio changed over the years. The odds of not reporting the method for the three predictors, and the impact of the FDA draft guidance on the odds of using the fixed-margin method were assessed using logistic regression.

A sensitivity analysis was performed using all margins that were identified in the included articles. A proportion of the included trials used more than one non-inferiority margin to analyze non-inferiority for one or more outcomes. The sensitivity analysis was conducted for the primary outcome using a Poisson model using all margins with reported methods. Nevertheless, the effect of clustering was ignored. Similarly, the clustering was ignored in the sensitivity analysis of the secondary outcome that was conducted using all identified margins. The sensitivity analyses of the three predictors and the use of the fixed-margin method using all reported margins were conducted using the generalized estimating equations with a logit link and an exchangeable correlation matrix. All statistical analyses were performed using IBM® SPSS® Statistics for Windows Version 23.0 (IBM Corp., Armonk, NY, USA). This review was reported according to PRISMA (Additional file 1: PRISMA checklist).

## Results

### Identification of articles in the systematic review

**Fig. 1:**
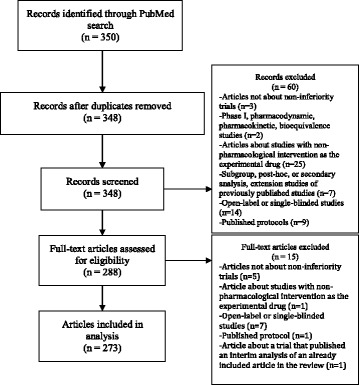
Flow chart of the search and screening process

The PubMed search yielded 350 abstracts. After screening these abstracts using our inclusion and exclusion criteria, the full texts of 288 articles were retrieved and assessed for eligibility. The selection and exclusion processes are illustrated in a PRISMA flow diagram (Fig. 1). The screened articles were published in the period from 2000 to 2015. A bibliography of the finally included articles in this review is provided as a supplementary material. We finally included 273 articles that account for 273 primary non-inferiority margins. Some articles reported a pooled non-inferiority analysis for two or more trials (we considered them as one trial). Thirty-seven trials (13.5%) used two or more margins to assess non-inferiority for one or more outcomes; the total number of margins identified was 326.

### Primary and secondary outcomes

**Fig. 2:**
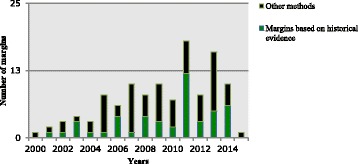
The number of margins defined by other methods versus the historical evidence over time

**Fig. 3:**
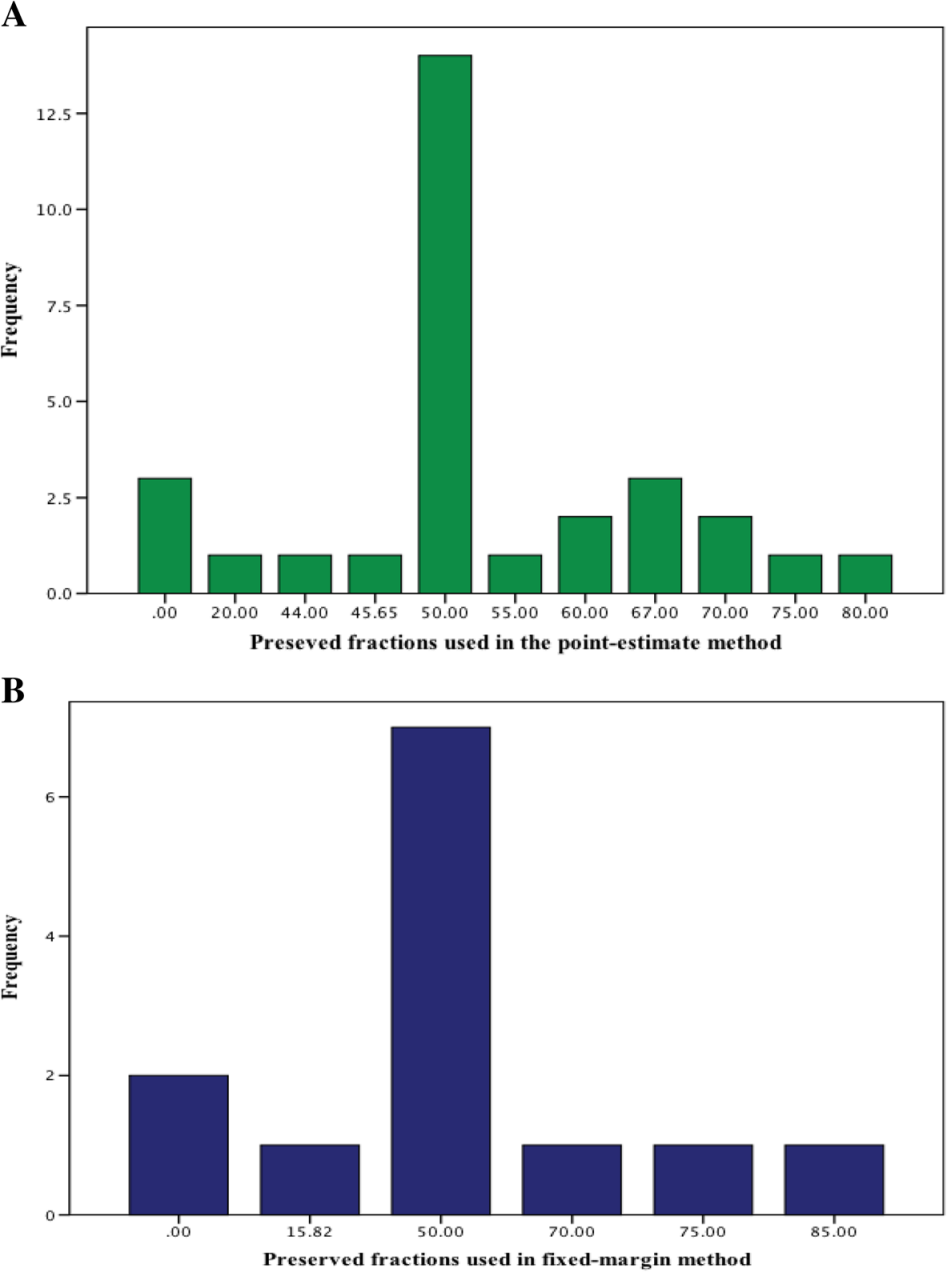
The range of preserved fractions used in the point-estimate method (**a**) and in the fixed-margin method (**b**)

Information about how the margin was defined was available for only 115 of 273 margins (42.1%). Among these 115 margins, the historical evidence of the active comparator was used to define 47 margins (40.1%), and 68 (59.9%) were defined by other methods (Table 1). The ratio of the number of margins that were defined by the other methods compared to the number of margins that were defined by historical evidence of the active comparator (ratio 2.17, 95% CI 0.86 to 5.82, *p* = 0.11) did not change over the years (i.e., no significant interaction between the method and the year variables) (Fig. 2). The preserved fractions that were used in the historical evidence methods ranged from 0 to 85% (Fig. 3).

**Table 1:** Classification of other methods used to define the margins in the systematic review

Subtype	Definition	Frequency (*n* = 68)
Expert opinion	The non-inferiority margin was chosen based on expert opinion. It also includes defining the margin based on: (1) the Delphi approach, (2) a threshold (e.g., superiority margin) for clinical efficacy that was chosen from the literature and considered by the authors, researchers, or experts as a clinically acceptable non-inferiority margin	42 (62%)
Based on literature review	1. Non-specific literature review: the choice of the margin was based on a review of the literature without indicating how the review was conducted, and what types of historical data were reviewed	3 (5%)
	2. Based on historically controlled data: the margin was defined based on the assessment of historical experience from non-concurrently controlled trials. The relative efficacy of the historical experience of active comparator was assessed against a historical group (e.g., placebo group, spontaneous cure rate group, or outcome without treatment group)	2 (3%)
	3. Review of other types of literature: the margin was defined based on the assessment of other types of literature (e.g., observational studies)	2 (3%)
The margin was used in other non-inferiority trial(s) with similar design	A similar margin was used in other non-inferiority trial(s) of drugs that are used to treat the same indication, regardless of whether the active comparator was used in these trials or not	9 (13%)
Regulatory consultation/guideline	The choice of the margin was based on one of the following: recommendations by a regulatory authority, following a guideline from a regulatory authority without indicating how the margin was exactly defined, or used a margin that is provided by one of the regulatory guidelines without indicating how exactly it was defined (neither by the authors nor by the guideline)	5 (7%)
Based on the efficacy of the experimental drug from the previous clinical trials	The margin was defined based on the efficacy of the experimental drug itself from the previous trials	5 (7%)

**Fig. 4:**
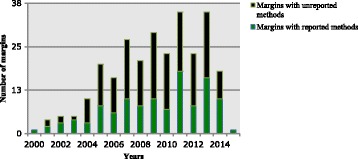
The number of margins with unreported methods versus reported methods over time

The method was not reported for 158 of 273 margins (57.9%). The ratio of the number of margins with unreported methods (*n* = 158) compared to the number of margins with reported methods (*n* = 115) (ratio 1.35, 95% CI 0.76 to 2.43, *p* = 0.31) did not change over time (i.e., no significant interaction between the reporting of the method and the year variables) (Fig. 4).

The results of the sensitivity analysis for the primary, secondary, and other outcomes were consistent with the results of the original analyses (supplementary materials).

### Predictors for reporting the methods of defining the non-inferiority margin

The odds of not reporting the method of defining the margin after versus before the publication of the 2006 extension of the CONSORT Statement were comparable (OR 1.08; 95% CI 0.61 to 1.91, *p* = 0.80). Also, the 214 (78.4%) margins reported in CONSORT-endorsing journals and the 59 (21.6%) margins reported in non-CONSORT-endorsing journals did not differ regarding the under-reporting of the method (OR 0.77; 95% CI 0.42 to 1.39, *p* = 0.4). However, the method for defining the margin was less often reported in low-impact journals in comparison to high-impact journals (OR 0.20; 95% CI 0.10 to 0.37, *p* < 0.0001).

### The impact of the FDA draft guidance on the use of the fixed-margin method

Thirty-four of 273 margins were defined based on the historical evidence of the active comparator using the effect estimate (33 were used to analyze non-inferiority by the point-estimate method, and one by the synthesis method). Thirteen margins of 273 (4.8%) were defined based on the limit of the confidence interval that is the closest to the null effect (the fixed-margin method). The publication of the FDA draft guidance in 2010 was associated with increased reporting of the fixed-margin method (after versus before 2010) (OR 3.54; 95% CI 1.12 to 13.35, *p* = 0.04). The use of this method increased from 4/163 (2.5%) before to 9/110 (8.2%) after the publication of the guidance.

## Discussion

We have not found a trend of an increase in the use of the historical evidence to define the non-inferiority margin compared to other methods. Even though the number of margins that were defined by the fixed-margin method increased after the publication of the FDA draft guidance, the proportion of these margins is very small. We also observed that the rationale for the choice of the non-inferiority margin is often not reported without a trend of improvement. As for predictors, only the publication in high-impact journals was associated with a better reporting of the methods of defining the margin.

The proportion of the margins in this systematic review that were defined based on historical evidence (17.2% of 273 trials) was larger than the proportion that was found by Lange and Freitag (8% of 314 trials) [19]. The latter review was the only review that provided details on how the data of the active comparator were exactly used to define the margin. Most of the other reviews reported whether the margin was defined based on “clinical or statistical considerations”, or whether it was defined based on “clinical and statistical considerations or results of a previous study” [8–11]. We found a wide range of preserved fractions used (0 to 85%), with a median of 50%. This median preserved fraction is in between the median preserved fractions found by Lange and Freitag (less than 50%) and by Wangge et al. (greater than 50%) [9, 22].

The rationale for the choice of the non-inferiority margin was not provided for more than half of the margins in this systematic review. This is consistent with the range of under-reporting of the rationale in previous reviews (54.3 to 84.7%) [8–12, 22, 23]. Our results are also comparable to the results of previous systematic reviews with regard to the insignificant impact of the extension of the CONSORT Statement on the reporting of the rationale for the choice of the margin so far. Additionally, we found no difference in reporting the rationale between CONSORT-endorsing and non-CONSORT-endorsing journals. Nevertheless, the extension was published in 2006 and was updated in 2010, it may be too early for the extension to have a significant impact on reporting non-inferiority trials in general, and on the rationale for the choice of the margin in particular. Indeed, the endorsement of the CONSORT Statement has improved the quality of reporting of randomized controlled trials [24]. For example, the results of a Cochrane review of 50 evaluation reports of more than 16,000 randomized controlled trials showed that the quality of reporting clinical trials was better in CONSORT-endorsing journals [24]. Finally, and in contrast to the results of the review by Wangge et al. where no difference was found in reporting the rationale between high- and low-impact journals [9], the publication in high-impact journals was associated with better reporting of the methods used to define the margin.

Among the 115 margins with reported methods, 36.5% were defined based on expert opinion. This is in line with the range of margins that were defined by expert opinions in previous reviews (25 to 75%) [9, 11, 22, 23]. Our findings also highlight the issue of not providing enough details on the method that was used to define the margin. Nine margins were chosen because they were used in similar non-inferiority trials, and five were defined based on regulatory recommendations or guidelines. Moreover, three margins were defined based on literature reviews without showing the review process or the type of literature that was reviewed.

Almost 60% of the margins identified in this review were used without reporting why and how they were selected. This may partially be due unfamiliarity with methods of defining the margin. This is supported by the review by Wangge et al. of the regulatory scientific advice on non-inferiority trials, which showed that 28% (98 of 354) of the questions and positions about non-inferiority trials that were sent by the pharmaceutical companies to the European Medicines Agency (EMA) were related to the margin [25]. Of the 86 responses to these questions, the EMA recommended stricter margin in 35 responses (41%) and questioned the justification of the margin in another nine (10%) responses. Importantly, lack of clarity on how the margins were arrived at may lead to incorrect decisions made by regulators, health technology assessment organizations, and health care professionals, and ultimately suboptimal health care.

Another contributing factor to the under-reporting of the rationale is that editors and journals do not strictly request reporting the exact method that was used to define the margin. Only two (1.7%) of the 118 journals state clearly that authors must follow the extension of the CONSORT Statement for non-inferiority trials if they are submitting a non-inferiority trial. Editors must realize that they are accepting non-inferiority trials that have suboptimal or unclear outcomes because the results of these trials were analyzed based on unmotivated non-inferiority margins. Journals should not be accepting non-inferiority trials without providing the rationale for the choice of the margin and should put a strong emphasis on following the extension of the CONSORT Statement itself when reporting non-inferiority trials.

In contrast to previous systematic reviews, our systematic review provides a detailed description of the methods being used to define non-inferiority margins, the impact of the FDA draft guidance on the use of the fixed-margin method, and the reporting of the rationale for the choice of the margin in CONSORT-endorsing and non-CONSORT-endorsing journals. However, our study has several limitations. First, for 158 of the 273 margins the method to define the margin was not reported. Therefore, we could only use 115 margins to evaluate for which percentage historical evidence was used to define the margin. The only predictor of reporting the method to define the non-inferiority margin was the impact factor (high versus low) of the journal in which the article was published. Under the assumption of missing data being missing at random (MAR), we also assessed the percentage of non-inferiority margins that are based on historical evidence stratified by impact factor, which were found to be 49% and 36% among high- and low-impact factor journals, respectively. The contribution of high- and low-impact journals is 58 and 215, respectively, leading to an estimate of the overall percentage of margins that are based on historical evidence of 39% (accounting for missing data), which does not materially differ from the 41% mentioned previously. Second, we might have missed some non-inferiority trials in our search, because different terms for “non-inferiority” might have been used or trials may have been published in journals that are not indexed in PubMed. These might also be the reasons for not detecting non-inferiority trials before 2000 in our review. However, in a review that was conducted to review changes in the publication of non-inferiority trials over 20 years, only one published non-inferiority trial was identified between 1989 and 1998 [1]. In that review, the search was conducted in both PubMed and EMBASE for all randomized non-inferiority trials. This provides assurance that we have not missed a large number of trials before 2000. Finally, we have not included open-label non-inferiority trials in our study, which might have provided broader evaluation of the methods of defining non-inferiority margins.

## Conclusions

In conclusion, methods of defining non-inferiority margins are not well reported. Data of those that are reported showed that margins is not commonly defined based on the historical evidence of the active comparator as recommended by regulators, and this, besides the reporting of the method, has not improved over recent years. Authors, reviewers, and editors need to take notice of these issues to allow for better judgment of non-inferiority trials.

## Additional file

Additional file 1: PRISMA 2009 Checklist (DOC 64 kb)

## Abbreviations

CONSORT: Consolidated Standards of Reporting Trials
EMA: European Medicines Agency
FDA: US Food and Drug Administration
ICMJE: International Committee of Medical Journal Editors
JCR: Journal Citation Reports
